# Prediction of spin–spin coupling constants with machine learning in NMR

**DOI:** 10.1002/ansa.202000180

**Published:** 2021-03-20

**Authors:** Kaina Shibata, Hiromasa Kaneko

**Affiliations:** ^1^ Department of Applied Chemistry School of Science and Technology Meiji University Kawasaki Japan

**Keywords:** LightGBM, machine learning, NMR, regression, spin–spin coupling constant

## Abstract

Nuclear magnetic resonance (NMR) spectroscopy is one of the most important methods for analyzing the molecular structures of compounds. The objective in this study is to predict indirect spin–spin coupling constants in NMR based on machine learning. We propose important descriptors for predicting indirect spin–spin coupling constants from target pairs of atoms in molecules, and combine the proposed descriptors with molecular descriptors to predict indirect spin–spin coupling constants with LightGBM as a regression analysis method. We construct regression models using a dataset and verify their predictive accuracy, and then confirm that the proposed descriptors can predict indirect spin–spin coupling constants more accurately than the traditional descriptors used to predict chemical shifts.

## 1 INTRODUCTION

Nuclear magnetic resonance (NMR) spectroscopy is one of the most important methods for analyzing the molecular structures of compounds. NMR is a spectroscopic technique in which a sample in a magnetic field is irradiated with constant radio waves to observe its spectrum, enabling structural analysis and quantitative measurements of compounds.^1^, ^2^ Most NMR experiments are performed with radio frequency pulses and Fourier transform. Because it provides useful information on intermolecular and intramolecular interactions and molecular dynamics, in addition to molecular structures, NMR is used in a wide range of fields such as chemistry, materials science, medicine, and life sciences.

NMR measurements provide information on chemical shifts, signal intensity, and indirect spin–spin coupling constants. Chemical shifts refer to the resonance frequencies of specific nuclear spins within molecules, and their values are determined by the environment around the nucleus. These are one of the most important components in the identification of molecular structures using NMR. The signal strength can be calculated from the intensity of the NMR spectra, and provides information on composition ratios and mixing ratios. From indirect spin–spin coupling constants, information on the relative bond distances and angles within molecules can be extracted using relationships between indirect spin‐spin coupling constant and geometrical parameters, and some relationships are found empirically and others can be provided by theoretical quantum chemistry. Then, this information is useful in determining the bonds between atoms and the dihedral angles of molecules in stereochemistry. Scalar coupling or *J* coupling is an indirect interaction between the nuclear spins of two atoms in a magnetic field. The number that comes before the *J* in the *J* coupling types (1*J*, 2*J*, 3*J*) denotes the number of bonds between the atoms that are coupling. For example, ^1^ *J*_CH_ is the spin–spin coupling constant between a hydrogen atom and a carbon atom separated by one bond (or simply bonded).

To investigate whether the chemical structure of a synthesized compound is consistent with the target structure, NMR results have been predicted from compound structures.^3^ The above three pieces of information obtained from NMR can be predicted by ab initio quantum chemistry methods from which the density functional theory (DFT) is by far the most computationally efficient.^4^, ^5^, ^6^ However, the DFT calculations are computationally intensive. When molecular designs are performed with virtual screening in chemoinformatics and indirect spin–spin coupling constants are used as molecular descriptors in virtual screening, it is required to calculate indirect spin–spin coupling constants for a huge number of molecules, and thus, it is necessary to calculate indirect spin–spin coupling constants fast. To overcome this problem, machine learning can be used as an alternative to DFT calculations. Using suitable datasets, regression models of the form *y *= f(*X*) are constructed between the descriptors of chemical structures, *X*, and the NMR results, *y*, and then the *y*‐values can be predicted from the *X*‐values of samples whose *y*‐values are unknown. Regression models can predict *y*‐values in a shorter time than DFT calculations. It is currently possible to predict chemical shifts from three‐dimensional chemical structures through machine learning,^7^, ^8^, ^9^, ^10^ and Gerrard et al predicted ^1^ *J*_CH_ coupling using similarity between chemical environments of each molecular structure and machine learning.^11^ Sahakyan predicted eight types of scalar coupling with machine learning using descriptors of the distance between the given points of each atom, the number of bonds on a specific atom, the square root of the sum of the squared Cartesian values, and the difference between the electronegativities of two atoms,^12^ however, whole chemical structures for molecules are not considered.

The objective of this study is to construct regression models that predict indirect spin–spin coupling constants considering whole chemical structures for molecules. We propose descriptors containing important information on indirect spin–spin coupling constants from three‐dimensional chemical structures, and then construct regression models between the proposed descriptors and the indirect spin–spin coupling constants. The regression models can be used to predict indirect spin–spin coupling constants for new chemical structures. In this study, we demonstrate the effectiveness of the proposed method using a Kaggle dataset.^13^

## 2 MATERIALS AND METHODS

We use a dataset of the first version of champs‐scalar‐coupling^13^ downloaded from Kaggle^14^ that consists of three‐dimensional chemical structures and indirect spin–spin coupling constants for 85003 molecules. The molecules contained only the atoms: carbon (C), hydrogen (H), nitrogen (N), fluorine (F), and oxygen (O). Because indirect spin–spin coupling constants exist for two atoms in a molecule, there are 4 658 147 sets of spin–spin coupling constant and target atom pairs in the current dataset. The dataset contains data for eight types of scalar coupling: ^1^ *J*_NH_, ^1^ *J*_CH_, ^2^ *J*_HH_, ^2^ *J*_NH_, ^2^ *J*_CH_, ^3^ *J*_HH_, ^3^ *J*_CH_, and ^3^ *J*_NH_, and the numbers of samples are 43363, 709416, 378036, 119253, 1140674, 590611, 1510379, and 166415, respectively.

In this study, we construct regression models of the form *y *= f(*X*) in which *y* is the spin–spin coupling constant between a target pair of two atoms and *X* is descriptors that provide numerical representations of the target pair of two atoms. Traditional descriptors used to predict chemical shifts from three‐dimensional chemical structures^7^ did not consider distances between atoms. Similarity between chemical environments of each molecular structure^11^ can only be combined with regression analysis methods based on the kernel method. The proposed descriptors *X* for a pair of two atoms are listed in Table 1. The molecular descriptors computed in RDKit (version 2019.09.1),^15^, ^16^ which is an open‐source library used in the field of chemoinformatics, are also used in this study, as they were used in the prediction of chemical shifts with machine learning^7^; examples of these descriptors include the number of bonds of the target atom, the presence or absence of rings, hybridization orbitals, aromaticity, the number of atoms in a molecule, the number of bonds, and the molecular weight. Bond angles and dihedral angles would be considered in the molecular descriptors provided by RDKit. The descriptors derived from the Euclidean distance between atoms with the same type of scalar coupling in a molecule include the mean, maximum, minimum, and standard deviation, and the difference and quotient of their values. The Euclidean distances between the midpoint of a target atom pair and the closest oxygen/nitrogen atom in a molecule are taken as distance descriptors. When there are no oxygen atoms in a molecule, the distance is set to 1000. The same is true in the absence of nitrogen atoms.

**TABLE 1:** Proposed descriptors for prediction of indirect spin–Spin coupling constants

Descriptors	Number
Molecular descriptors calculated with RDKit,^15^, ^16^ eg, hybridization orbitals and aromaticity	210
Euclidean distance between atoms	1
Descriptors derived from Euclidean distance between atoms with the same type of scalar coupling in a molecule, eg, average of Euclidean distance between atoms with the same type of scalar coupling	49
Euclidean distance between the midpoint of a target atom pair and an oxygen atom in a molecule, and Euclidean distance between the midpoint of a target atom pair and a nitrogen atom in a molecule	12
Euclidean distance between a target atom and an oxygen atom in a molecule, and Euclidean distance between a target atom and a nitrogen atom in a molecule	24
Total	296

Because both target atoms are hydrogen atoms in the case of ^2^ *J*_HH_ and ^3^ *J*_HH_ types of scalar coupling, the descriptors are based on the distances between target atoms that are the same, and so the distances between target atoms and oxygen/nitrogen atoms are deleted. In their place, the average distance between the target hydrogen atom and its nearest carbon or nitrogen atom, and the average of the mean, minimum, maximum, and standard deviation calculated are added as descriptors. We did not select the descriptors based on correlation between descriptors and some importance in this study.

We use LightGBM,^17^ which is one of the methods with highly predictive ability, as a regression analysis method based on a decision tree and ensemble learning and the Python library of the reference^18^ whose version is 2.3.0. The LightGBM parameters are presented in Table 2.

**TABLE 2:** Parameters in LightGBM

Parameter	Value
num_leaves	128
min_child_samples	79
bagging_seed	11
learning_rate	0.2
reg_lambda	0.3
boosting_type	gbdt
subsample_freq	1
Subsample	0.9
Objective	regression
Metric	mae
verbosity	−1
reg_alpha	0.1
max_depth	13
colsample_bytree	1.0

## 3 RESULTS AND DISCUSSION

In this study, the data were randomly divided into a training set containing 59 502 molecules (70%) and a test set containing 25 501 molecules (30%). Since the numbers of both training samples and test samples were high and the molecules were randomly divided, diverse chemical structures were included in both training samples and test samples. Regression models were constructed from the training data for each type of scalar coupling, and *y*‐values were predicted for the test data. The RDKit descriptors of which values are 95% same were removed. For the ^1^ *J*_NH_, ^2^ *J*_NH_, and ^3^ *J*_NH_ types of scalar coupling, the distance to the nitrogen atom closest to the target atom is zero, and so this descriptor was removed.

Using LightGBM, the proposed method was compared with the traditional method, in which the descriptors are the RDKit descriptors used for chemical shift prediction^7^ and the Euclidean distance between the target atoms. The prediction results for the test data for each type of scalar coupling are presented in Table 3, where *r* ^2^ is the determinant coefficient (higher values denote better predictive accuracy), *RMSE* is the root‐mean‐squared error (lower values denote better predictive accuracy) and *RRMSE* is the relative RMSE in which RMSE is divided by the standard deviation of each *y*. As shown in Table 3, for all types of scalar coupling, the proposed method gives higher *r* ^2^ and lower *RMSE* values than the traditional method, indicating the improved predictive accuracy of the proposed method. In particular, the *RMSE* of ^2^ *J*_CH_ reaches ∼30%, with significantly reduced prediction errors. By adding the distances from oxygen and nitrogen atoms as descriptors, we can handle the contribution of oxygen and nitrogen atoms, which are more susceptible to polarity than hydrogen atoms, and the environment around carbon atoms, which bind with many types of atoms. The results confirm that the proposed descriptors work effectively.

**TABLE 3:** Prediction results for the test data. The traditional method means LightGBM models with traditional descriptors

	*r* ^2^		RMSE		RRMSE	
	Traditional method	Proposed method	Traditional method	Proposed method	Traditional method	Proposed method
^1^ *J*_NH_	0.977	0.992	1.65	0.98	0.151	0.090
^1^ *J*_CH_	0.959	0.990	3.70	1.82	0.203	0.100
^2^ *J*_HH_	0.903	0.986	1.25	0.48	0.312	0.120
^2^ *J*_NH_	0.858	0.981	1.39	0.51	0.378	0.139
^2^J_CH_	0.687	0.967	2.54	0.82	0.561	0.181
^3^ *J*_HH_	0.822	0.968	1.56	0.67	0.421	0.181
^3^ *J*_CH_	0.595	0.880	1.96	1.07	0.636	0.347
^3^ *J*_NH_	0.747	0.921	0.66	0.37	0.501	0.281

**FIGURE 1:**
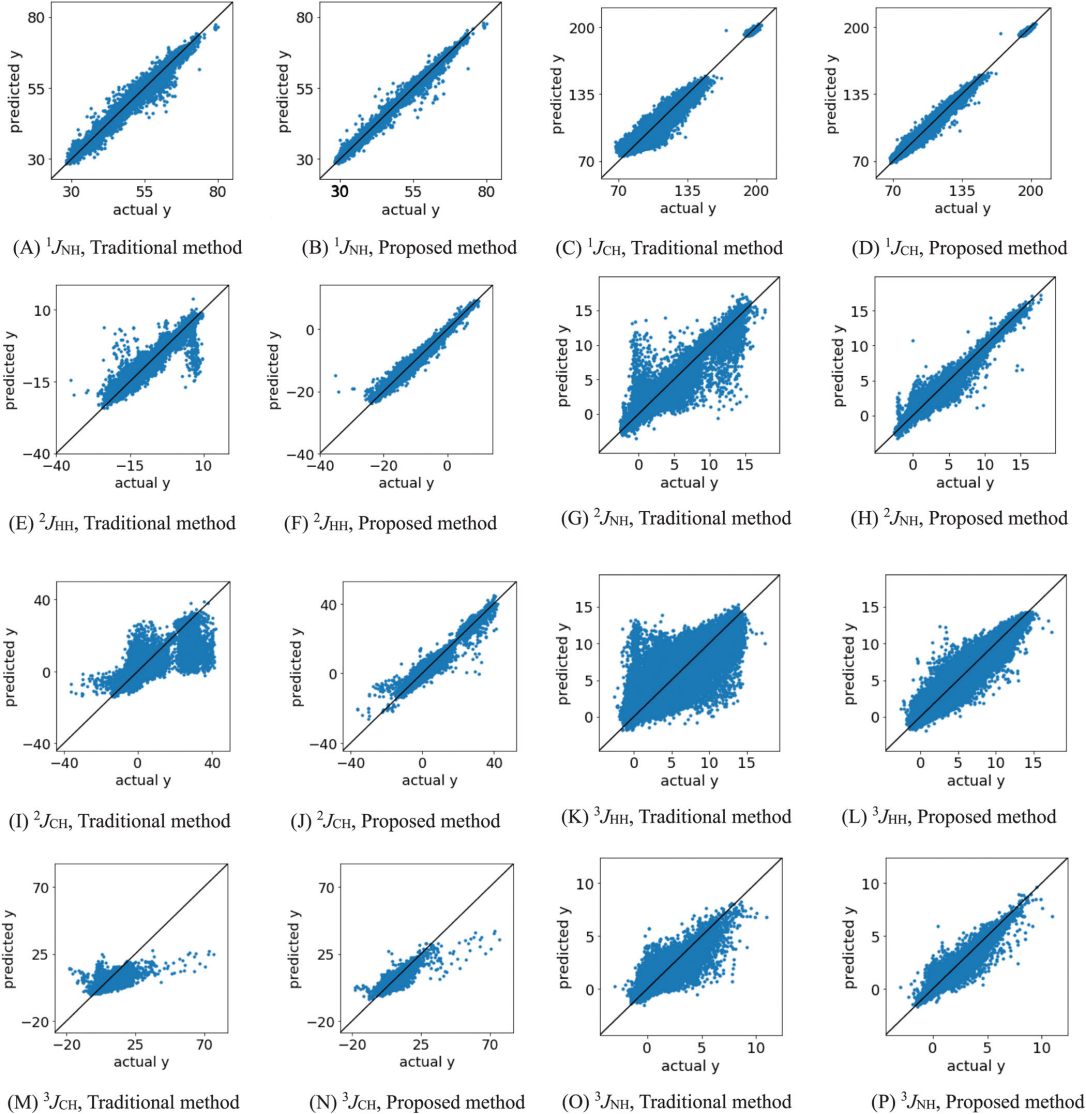
Actual *y*‐values vs. predicted *y*‐values with the traditional method and the proposed method for test data for eight types of scalar coupling: ^1^ *J*_NH_, ^1^ *J*_CH_, ^2^ *J*_HH_, ^2^ *J*_NH_, ^2^ *J*_CH_, ^3^ *J*_HH_, ^3^ *J*_CH_, and ^3^ *J*_NH_

Figure 1 shows the plots of actual *y*‐values versus predicted *y*‐values in the test data for each type of scalar coupling. For all types of scalar coupling, the samples given by the proposed method are closer to the diagonal than those given by the traditional method, indicating that the proposed method predicts the indirect spin–spin coupling constants with high accuracy. This confirms that the proposed descriptors can effectively predict the indirect spin–spin coupling constants.

For each type of scalar coupling, descriptors with the highest variable importance in LightGBM are listed in Table 4. For all types of scalar coupling, the descriptors related to the distance to the oxygen or nitrogen atom are in the top three, suggesting that it is useful to consider the environment from the target atom to the closest oxygen or nitrogen atom. For example, in ^2^ *J*_CH_, the distance between the target hydrogen atom and its nearest carbon or nitrogen atom, the distance between the midpoint of the target atoms and its nearest oxygen atom, and the distance between the target hydrogen atom and its nearest oxygen atom are the top three descriptors, indicating that information on the oxygen and nitrogen atoms around the target atom is important. In addition, in most cases, the atom having scalar coupling with the hydrogen atom is directly bonded to the hydrogen atom, and because this information is based on the distance between the hydrogen atom and the directly bonded atom, the distance from the atom to having scalar coupling with the hydrogen atom to the target hydrogen atom is considered to be the most important descriptor. However, the fact that some of the samples in the plots in Figure 1 are off the diagonal for each type of scalar coupling confirms that the environment around the atoms is not described sufficiently well.

**TABLE 4:** Descriptors with the highest variable importance in LightGBM for eight types of scalar coupling: ^1^ *J*_NH_, ^1^ *J*_CH_, ^2^ *J*_HH_, ^2^ *J*_NH_, ^2^ *J*_CH_, ^3^ *J*_HH_, ^3^ *J*_CH_, and ^3^ *J*_NH_

	Order	Descriptors
^1^ *J*_NH_	1	Euclidean distance between the target nitrogen atom and its nearest hydrogen, carbon, or nitrogen atom
	2	Euclidean distance between the target hydrogen atom and its nearest oxygen atom
	3	Euclidean distance between the target hydrogen atom and its second nearest nitrogen atom
^1^ *J*_CH_	1	Euclidean distance between the target hydrogen atom and its nearest oxygen atom
	2	Euclidean distance between the target carbon atom and its nearest oxygen atom
	3	Euclidean distance between the target atoms
^2^ *J*_HH_	1	Euclidean distance between the midpoint of the target atoms and its nearest oxygen atom
	2	Euclidean distance between the target atoms
	3	Average of the Euclidean distances between the target hydrogen atoms and their nearest carbon or nitrogen atoms
^2^ *J*_NH_	1	Euclidean distance between the target hydrogen atom and its nearest carbon or nitrogen atom
	2	Euclidean distance between the target atoms
	3	Euclidean distance between the midpoint of the target atoms and its nearest oxygen atom
^2^ *J*_CH_	1	Euclidean distance between the target hydrogen atom and its nearest carbon or nitrogen atom
	2	Euclidean distance between the midpoint of the target atoms and its nearest oxygen atom
	3	Euclidean distance between the target hydrogen atom and its nearest oxygen atom
^3^ *J*_HH_	1	Euclidean distance between the target atoms
	2	Euclidean distance between the midpoint of the target atoms and its nearest oxygen atom
	3	Average of the Euclidean distances between the target hydrogen atoms and their nearest carbon or nitrogen atoms
^3^ *J*_CH_	1	Euclidean distance between the target hydrogen atom and its nearest carbon or nitrogen atom
	2	Euclidean distance between the midpoint of the target atoms and its nearest oxygen atom
	3	Euclidean distance between the midpoint of the target atoms and its nearest nitrogen atom
^3^ *J*_NH_	1	Euclidean distance between the target hydrogen atom and its nearest carbon or nitrogen atom
	2	Euclidean distance between the midpoint of the target atoms and its nearest oxygen atom
	3	Euclidean distance between the target atoms

## 4 CONCLUSIONS

In this study, we proposed atom‐pair descriptors relating the distance between atoms, hybridization orbitals, and oxygen and nitrogen atoms around bonds for three‐dimensional molecular structures as a means of predicting the indirect spin–spin coupling constants between atoms. Regression models were constructed to predict indirect spin–spin coupling constants using the proposed descriptors and molecular descriptors as *X*‐variables with LightGBM. The proposed method was found to be more accurate than the traditional method used in predicting chemical shifts. Furthermore, the importance of the proposed descriptors was confirmed by calculating the most influential descriptors using LightGBM. Indirect spin–spin coupling constants predicted with the proposed method fast would be used as molecular descriptors in virtual screening. However, a limitation of the proposed method is the predictive accuracy compared with DFT calculation. In future work, the accuracy of the indirect spin–spin coupling constant predictions will be improved by adding descriptors that can deal with atoms other than oxygen and nitrogen atoms in the vicinity of the bond.

## CONFLICT OF INTEREST

The authors declare no conflict of interest.

## Data Availability

The data that support the findings of this study are available in ref. 13.
